# Daily Fungal Cell-Free DNA Testing to Assess Clinical Status during *Candida krusei* Fungemia

**DOI:** 10.3390/jof10070449

**Published:** 2024-06-27

**Authors:** Jo-Anne H. Young, Xiaoying Liu, Emma Porter, Hannah Sweet, Wei Wang, Anton F. Evans, Chi Zhang, Karam M. Obeid

**Affiliations:** 1 Division of Infectious Disease and International Medicine, Department of Medicine, University of Minnesota, Minneapolis, MN 55455, USA; kmobeid@umn.edu; 2 Zepto Life Technology, Inc., Saint Paul, MN 55114, USA

**Keywords:** cell-free DNA, cfDNA, point of care (POC), fungemia, *Candida krusei*, giant magnetoresistance (GMR)

## Abstract

We present a case of a man immunocompromised due to myelodysplastic syndrome with *Candida krusei* fungemia who had a rising cell-free DNA (cfDNA) giant magnetoresistance (GMR) signal when tested daily using plasma blood samples. With the rise in GMR signal paralleling the development of skin lesions in this patient, we conclude that cfDNA can be used to indicate uncontrolled infection and thus help monitor response to therapy. This index patient provides evidence that an invasive fungal infection requires both direct antifungal therapy and an intact immune system to control the infection. This biosensing platform has been simplified to potentially serve as a point-of-care test, setting it apart by overcoming the three common barriers of cfDNA testing: complexity, cost, and time.

## 1. Introduction

Invasive fungal infections (IFIs) represent a severe health threat to vulnerable patient populations, such as those residing in intensive care units and those with compromised immune systems [1]. As a group, invasive candidiasis, aspergillosis, and cryptococcosis account for the majority of these IFIs [1]. The morbidity and mortality rates associated with these IFIs are alarmingly high, particularly when these infections are diagnosed in the late stages of infection, leading to significant complications and death. Physicians should suspect these infections among the appropriate hosts and be aggressive in their diagnoses and treatments [2,3,4,5].

Early diagnosis as well as monitoring the response to therapy are both critical for effective patient management [6]. Plasma cell-free DNA (cfDNA) testing can improve the detection of fungal pathogens in body fluid samples, particularly in providing rapid test results when traditional methods cannot deliver timely information [7,8]. Commercially available cfDNA testing methods, including real-time polymerase chain reaction (PCR), digital PCR, and next-generation sequencing (NGS), may not always have the combination of desired test attributes, which include high sensitivity (<50 copies/mL) and specificity, capability for multiplex detection of multiple fungal targets, complete automation, rapid turnaround time, bedside testing, and cost-effectiveness [9]. 

Giant magnetoresistance (GMR) is a quantum mechanical magnetoresistance effect observed in multilayers composed of alternating ferromagnetic and non-magnetic conductive layers [10,11,12,13,14,15,16,17]. The coupling of the GMR sensors with biological markers results in extremely sensitive assays. Reported studies have shown GMR-based assays can detect as few as 10 copies per mL of biological markers such as pathogenic DNA [18,19,20], making them highly effective for medical diagnostics. In this study, we used GMR to detect trace amounts of PCR amplicons in cfDNA. Concerning cost and automation, GMR technology stands out because it can potentially integrate all the necessary components onto a single cartridge, reducing the need for multiple separate procedures and thereby lowering the overall cost of laboratory testing. The rapid turnaround and reduced cost also make this platform desirable for laboratory diagnosis and treatment monitoring for IFIs. 

Our center is testing GMR technology as a high-sensitivity, cost-effective solution for detecting multiple fungal targets simultaneously. Our local Institutional Review Board approved a study testing patients’ blood samples in parallel with standard-of-care monitoring and treatment of fungal infections. In one of the cases, we noted a rise in the *Candida krusei* (*C. krusei*) cfDNA GMR signal for a patient receiving antifungal treatment for *C. krusei* fungemia, even after the resolution of fungemia. In this article, we report the results of the lookback into this patient’s condition for an explanation for the rise in fungal cfDNA. 

## 2. Materials and Methods

Approval for the collection of specimens was obtained by the University of Minnesota Institutional Review Board under study protocol #00020540. Whole blood was saved daily in 3 mL EDTA vacutainer tubes (Greiner Bio-One round bottom polystyrene Vacuette^®^ tubes, Monroe, NC, USA) from patients with body fluid cultures from one of 16 fungal organisms on the GMR test panel (*Candida albicans*, *C. auris*, *C. glabrata*, *C. krusei*, *C. tropicalis*, *C. parapsilosis*, *Aspergillus flavus*, *A. fumigatus*, *A. niger*, *A. terreus*, *Cryptococcus neoformans*, *Fusarium verticillioidis/oxysporum*, *F. solani*, *Histoplasma*, *Coccidioides immitis/posadasii*, *Pneumocystis jirovecii*). Sample tubes were stored upright for 1–4 days at refrigerator temperature (4 °C). Sample tubes were transported within 1 h in a cooler to the Zepto Life Technology Inc. (Zepto, St Paul, MN, USA) laboratory. Upon receipt, plasma was isolated from whole blood samples by centrifugation at 1500× *g* for 15 min. Plasma was then transferred to sterile 1.5 mL tubes and stored at −80 °C until testing was conducted. 

CfDNA was extracted using the MagMax cfDNA isolation system (Applied Biosystems, Waltham, MA, USA) from plasma samples following digestion with proteinase K. The resulting purified cfDNA was subdivided and used as a template for a series of multiplexed PCR reactions. The resulting amplicons were combined and selectively digested using an exonuclease.

The digested PCR amplicon products were then made to flow over a 36-plex GMR sensor, which is surface-coated with oligonucleotide probes specific to different target molecular targets and used to determine the specificity of the fungal species. As the sample flows over the sensor, the digested amplicons can selectively hybridize with the probes. After hybridization, magnetic beads are introduced, which bind selectively to the amplicon–probe complexes. The magnetic beads produce a detectable GMR signal specific to each sensor, with the signal strength corresponding to the variable levels of hybridized complexes on the GMR sensor surface. This, in turn, allows for an approximation of comparative fungal cfDNA levels within a patient’s sample. 

## 3. Case Patient

The index fungal infection was *C. krusei* fungemia in a 66-year-old neutropenic man undergoing chemotherapy with daunorubicin and cytarabine due to relapsed myelodysplastic syndrome (MDS), which had progressed into acute myeloid leukemia (AML). The fungemia occurred while on a prolonged course of prophylactic posaconazole. Due to the drug–drug interaction between posaconazole and cytarabine, micafungin was used as a temporary alternative to posaconazole during chemotherapy cycles.

**Figure 1:**
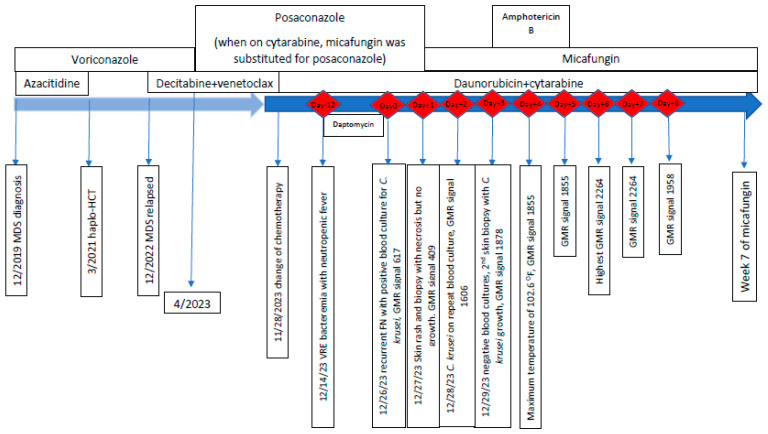
Timeline of the patient’s myelodysplastic syndrome, leukemia history, and index fungemia. The timeline arrow in light blue represents the history before admission during which the index fungemia occurred. The timeline arrow in darker blue represents the admission during which the index fungemia occurred. *C. krusei: Candida krusei*; GMR: giant magnetoresistance; Haplo-HCT: haploidentical allogeneic hematopoietic cell transplant; MDS: myelodysplastic syndrome; VRE: vancomycin-resistant *Enterococcus faecium*.

The patient experienced a neutropenic fever. The blood cultures grew vancomycin-resistant *Enterococcus faecium* (VRE), which was treated with daptomycin. The peripherally inserted central catheter (PICC) was removed. Twelve days later, while on daptomycin, neutropenic fever recurred (day 0 in Figure 1), and blood cultures were obtained at four time points (4:54 am, 04:59 am, 6:18 pm, and 6:42 pm). The following day (day+1), all four blood cultures identified budding yeast, so fungal coverage was changed from posaconazole to micafungin 100 mg daily (1.5 mg/kg). The yeast was speciated to *C. krusei* on day+2 and blood cultures were repeated at 05:46 am. The repeat blood cultures grew yeast on day+3, which was speciated to *C. krusei* on day+4.

The recurrent neutropenic fever and the fungemic episode were associated with abdominal pain. Computed tomography (CT) imaging showed no intra-abdominal fluid collection, colitis, or any other focus of fungal infection. The translocation of gut flora, including yeast and bacteria, was presumed to be the most likely etiology of the fungemia due to prolonged neutropenia, abdominal pain, and VRE bacteremia preceding the *C. krusei* fungemia.

**Figure 2:**
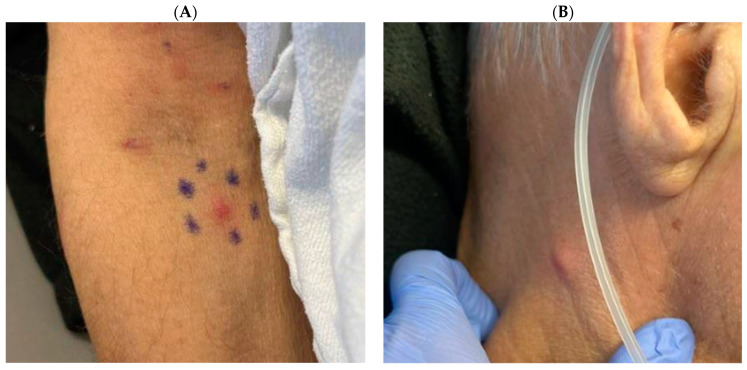
Photographs of skin lesions from this index case patient: panel (**A**): skin lesions located on the right arm and in the right antecubital area; panel (**B**): skin lesion located on the right side of the neck.

A pink, nodular, non-blanching, non-painful rash was seen on day+1; photographs are in Figure 2. A skin biopsy showed deep dermal and subcutaneous fibrosis with focal necrosis, suspicious for an angioinvasive fungal process. Fungal stain and culture were negative, however. On day+3, due to progressive rash and persistent fever, a skin biopsy was repeated, the dose of micafungin was increased to 150 mg daily (2.3 mg/kg), and liposomal amphotericin B was added at 350 mg daily (5 mg/kg). Fungal culture from the second skin biopsy grew yeast, which was identified as *C. krusei* on day+5.

Further studies included a transthoracic echocardiogram with no evidence of valvular structural abnormalities or vegetation on day+3 and a dilated funduscopic eye examination that did not show endophthalmitis or retinitis on day+4. By day+5 the patient reported feeling better with an improving rash, so liposomal amphotericin B was discontinued after two complete doses. 

Due to prolonged neutropenia, the patient had prolonged antifungal drug exposure to posaconazole alternating with micafungin during the 7 months before the index fungemia. During that period, numerous posaconazole blood levels were all therapeutic, ranging from 1.1 to 3.1 μg/mL (therapeutic range, 0.7 to 5.0 μg/mL). Hence, a titer-based, manual antifungal susceptibility testing was ordered on the bloodstream isolate, and on day+5, it returned susceptible to micafungin with a minimum inhibitory concentration (MIC) of 0.25 μg/mL. No interpretation was available for the other antifungal MICs, which included an MIC for posaconazole of 1.0 μg/mL, amphotericin B of 1 μg/mL, isavuconazole of 1 μg/mL, and voriconazole of 1 μg/mL. 

Blood cultures from day+3, +4, +5, and +6 were negative for yeast. The patient completed a total of 7 weeks of micafungin treatment, after which posaconazole was resumed for secondary fungal prophylaxis. 

## 4. Results

The GMR test results are presented in Table 1; these research tests were performed separately and not reported back to the clinical care team, so they did not influence patient care. There was a daily rise in signal, which paralleled the patient’s persistently elevated body temperatures, matched the clinical evolution of the skin lesions, and occurred while the patient remained heavily neutropenic. The peak of GMR occurred on day+6, two days following the peak temperature elevation on day+4. 

## 5. Discussion

*Interpretation of the GMR test signal.* In this case, we interpret that the persistent rise in the GMR signal, while the patient is on appropriate antifungal therapy and seemingly improving, is an indication of persistent infection. The development of skin nodules and the recovery of the yeast in the second skin biopsy confirmed persistent infection, even though blood cultures were without the growth of yeast. We followed the white blood cell count (WBC) as an indicator of the recovery of his immune system from chemotherapy. The first decline in the GMR signal paralleled the recovery of the immune system, further corroborating the importance of an intact immune system in controlling fungal infections. We believe that in addition to the clinical and immunologic recovery measured by the WBC, the GMR signal is an additional marker that helps guide the length of antifungal therapy, so it is neither prematurely discontinued nor continued for too long. This can be especially valuable if the GMR signal is available for point-of-care testing. 

*Current commercial cell-free DNA testing is complex*, *expensive*, *and not time-sensitive.* Diagnosis of human fungal infections using circulating free DNA, also called “cell-free” DNA, abbreviated as cfDNA, relies on the detection of degraded DNA fragments in body fluids. This testing is generally not simple since DNA has to be extracted from centrifuged body fluids, followed by sequence-specific detection. This process is generally performed at specialized facilities where human DNA can be separated from target fungal DNA, and the test is performed without contamination. Tests have to be transported to the facilities, often with controlled temperature needs, adding extra time between sample collection and test results. Additionally, this is specialized testing with expensive reagents and labor time, leading to high enough financial costs because the tests are performed infrequently. 

*Point-of-care testing in medical diagnosis.* When medical diagnostic testing is available at or near the time and place of patient care, it is referred to as point-of-care (POC) testing, near-patient testing, or bedside testing. This GMR biosensing platform has the potential to become a POC test for cfDNA, which makes it unique, bringing down the usual three barriers of current commercial cfDNA testing: complexity, cost, and time. 

*Validation of this POC cfDNA test.* Our center is evaluating this diagnostic method both early in the course of infection as well as for monitoring the response to therapy. A future stage of our evaluation will involve on-site testing with a unit in physical proximity to patient care; this is necessary for true POC, or near-care testing. This case demonstrates the value of early diagnosis as well as monitoring the response to therapy. Had this test been performed POC together with the initial blood culture, the diagnosis of *C. krusei* fungemia might have been known within an hour. The clinicians in this case thought they might be dealing with a bacterial infection and had only changed antibacterial therapy. The actual change in antifungal therapy occurred 24 h into the infection condition when the blood culture machine flagged this patient’s specimens as a yeast infection. 

*Role of GMR in discovering that a bloodstream infection has affected an end organ.* This patient had a change to micafungin 24 h into the course of the infection, but the GMR signal was rising when blood cultures cleared at 72 h. The clinical question that arose was what persisting source allowed cfDNA testing to remain positive, in contrast to blood cultures being positive only on day+0 and day+2. Since the estimated half-life of cfDNA in circulation is less than two hours (when assessed for tumor dynamics) [21], the cfDNA signal likely represented ongoing fungal infection despite negative blood cultures. A persisting source is often (1) an open portal of entry such as a mucosal ulcer in the gastrointestinal tract, (2) a persisting biofilm that has organisms embedded, or (3) a body site that harbors infection and needs either prolonged treatment or drainage in addition to the initial treatment. This episode of fungemia was likely due to a transient translocation of gut flora from chemotherapy, as the patient was not known to have any gut mucosal tears or ulcerations. A persisting biofilm was also unlikely, as the only long-term biologic hardware, a PICC line, had been removed five days before fungemia. In our patient’s example, the skin nodules were the body site harboring continued infection. We feel that a potential distant source that was seeded by the initial fungemia was effectively eliminated when clinical testing returned with an unremarkable cardiac echocardiogram and a negative dilated eye funduscopic examination. 

*Limitations*. The samples included in this study were rescued retrospective convenience samples obtained from leftover complete blood count (CBC) test K2EDTA tubes. This represents a limitation, as sample integrity may be compromised compared to prospectively collected samples. The opening of the tubes for CBC testing increases the risk of introducing environmental contaminants. Additionally, the time of sample storage varied from the time of draw to receipt and processing for testing.

*Conclusions.* We are cautious about this interpretation of the rise in the GMR signal. The quantitative nature of the assay is still being characterized, and we have to account for the effect PCR has on the amplification of the GMR signals. It is possible, though, that the skin nodular lesions, which harbored *C. krusei*, can account for the rise in the GMR signal.

## Figures and Tables

**Table 1:** Giant magnetoresistance testing results in relation to case–patient attributes.

	White Blood Count	Blood Culture	Skin Biopsy Culture	Maximum Fever	GMR Signal
Day+0	0.4 × 10^9^ cells/L	Positive for *C. krusei* at four time points		102 °F/38.9 °C	617
Day+1	0.3 × 10^9^ cells/L		Negative	101.1 °F/38.4 °C	409
Day+2	0.5 × 10^9^ cells/L	Positive for *C. krusei*		102.5 °F/39.2 °C	1606
Day+3	0.3 × 10^9^ cells/L	Negative	Positive for *C. krusei*	102 °F/38.9 °C	1878
Day+4	0.4 × 10^9^ cells/L	Negative		102.6 °F/39.2 °C	1855
Day+5	0.4 × 10^9^ cells/L	Negative		102.4 °F/39.1 °C	2112
Day+6	0.7 × 10^9^ cells/L	Negative		99.1 °F/37.3 °C	2264
Day+7	1.0 × 10^9^ cells/L			99.3 °F/37.4 °C	2264
Day+8	1.3 × 10^9^ cells/L			99 °F/37.2 °C	1958

Note: white blood count normal range is 4.0–11.0 × 10^9^ cells/L. F = Fahrenheit, C = Celsius. GMR = giant magnetoresistance. *C. krusei* = *Candida krusei*.

## Data Availability

The original contributions presented in the study are included in the article, further inquiries can be directed to the corresponding author.
